# Pneumomediastin spontané chez un asthmatique

**DOI:** 10.11604/pamj.2016.25.94.9957

**Published:** 2016-10-18

**Authors:** Amine Elmoqaddem, Issam Serghini, Hicham Janah, Chakib Chouikh, Amine Alaoui, Mustapha Bensghir

**Affiliations:** 1 Pôle d’Anesthésie Réanimation HMIMV Rabat, Maroc; 2 Service de Réanimation, Hôpital Militaire Avicenne Marrackech, Maroc; 3 Service de Pneumologie, Hôpital Militaire de Guelmim, Maroc

**Keywords:** Pneumomédiastin, asthme, dyspnée, Pneumomediastinum, asthmatic, dyspnea

## Abstract

Le pneumomédiastin spontané est une pathologie rare. Il se voit surtout chez l’adulte jeune. Sa survenue brutale est caractéristique, associant une douleur thoracique, un emphysème sous-cutané et une dyspnée. Nous rapportons l’observation d’un pneumomédiastin, chez un patient de 24 ans asthmatique déclenché suite à un effort de toux. Le patient s’est présenté dans un tableau d’insuffisance respiratoire aigue avec une douleur thoracique retrosternale. La radiographie pulmonaire a confirmé le diagnostic de pneumomédiastin. L’évolution a été favorable en 4 jours après exsufflation, oxygénothérapie et traitement médical conventionnel.

## Abstract (en)

Spontaneous pneumomediastinum is a rare disease. It especially affects young adults. The onset is usually sudden, involving chest pain, subcutaneous emphysema and dyspnea. We report the case of a 24-year old asthmatic patient with pneumomediastinum after coughing effort. The patient presented with acute respiratory failure associated with retrosternal chest pain. Chest X-ray confirmed the diagnosis of pneumomediastinum. The patient evolved favorably within 4 days after exsufflation, oxygen therapy and conventional medical treatment.

## Introduction

Le pneumomédiastin spontané est défini par la présence d’air au niveau du médiastin en dehors de tout contexte traumatique, iatrogène ou d’une maladie pulmonaire sous jacente. C’est une affection rare dont l’incidence est estimé à 1/32896 de la population générale [1]. Nous rapportons un cas de pneumomédiastin spontané chez un patient asthmatique.

## Patient et observation

**Figure 1:**
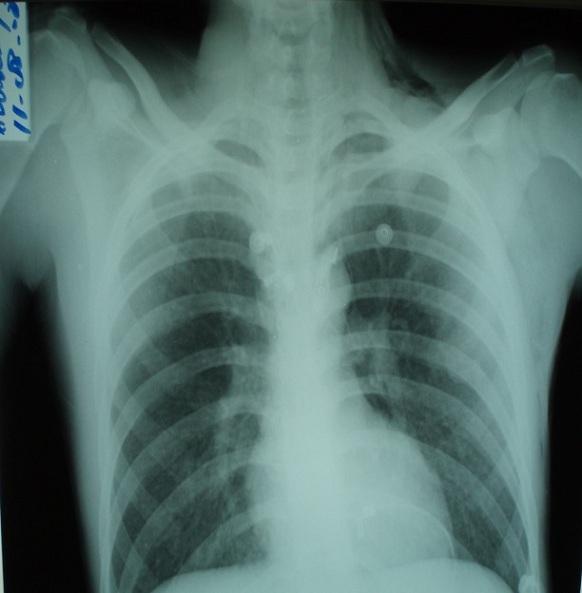
radiographie thoracique montrant le pneumomediastin: une fine hyperclarté linéaire silhouettant le bouton aortique, l’aorte descendante et le cœur

**Figure 2:**
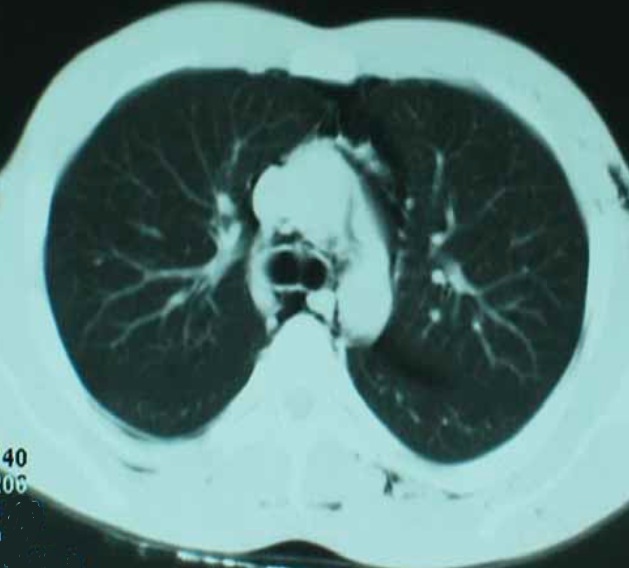
tomodensitométrie thoracique montrant l’extension du pneumomediastin vers les régions cervicales

Il s’agit d’un patient âgé de 24 ans, connu asthmatique admis aux urgences dans un tableau d’insuffisance respiratoire aigue. L’histoire de la maladie remonte à quatre jours par l’installation brutale d’une polypnée associée à une douleur thoracique retrosternale survenue un matin après un effort de toux. L’examen clinique trouve un patient polypnèique à 35 cycles par minute avec un balancement thoraco-abdominal et un tirage sus sternal et sous costal. L’auscultation pulmonaire trouve des râles sibilants, ainsi que des râles crépitants se projetant sur l’aire cardiaque (signe de Hamman). Des crépitations neigeuses basicervicale et thoracique antero-superieure signant la présence d’un emphysème sous cutané. La tension artérielle est de 130/70 mmHg. Les bruits du cœur sont normaux avec une fréquence à 110 battements par minute. La saturation en oxygène est à 85% à l’air ambiant et la gazométrie artérielle montre une hypoxémie à 80 mmHg. La radiographie pulmonaire objective une fine hyperclarté linéaire silhouettant le bouton aortique, l’aorte descendante et le cœur. Par ailleurs on note une extension du pneumomédiastin vers les régions cervicales et les parties molles cervico-thoraciques (Figure 1). Le scanner thoracique confirme la présence de l’air en péritrachéal, en péribronchique, en latero cardiaque et au niveau du médiastin antérieur (Figure 2). L’électrocardiogramme est normal et la Numération formule sanguine montre une éosinophilie à 5%. Le patient a été mis sous masque à oxygène à 5l/mn, de la ventoline en nébulisation et une corticothérapie par voie intraveineuse à raison de 80 mg de méthyle prédnisolone toutes les 8 heures. L’évolution en milieu de réanimation a été favorable après une exsufflation.

## Discussion

En 1618, le premier cas de pneumomédiastin spontané a été rapporté par Gordon, quand Louise Bourgeois avait observée un emphysème sous cutané chez une parturiente [1]. Puis secondairement décrit par Hamman en 1939 [2]. Le pneumomédiastin spontané, d’apparition le plus souvent brutale, se retrouve préférentiellement chez l’adulte jeune, de sexe masculin, ayant un morphotype longiligne [3–5]. Sa physiopathologie fait appel à un emphysème par la création d’un gradient de pression survenant lors des phénomènes d’hyperpression dans les alvéoles proches des septas vasculaires en périphérie des lobules (effet macklin) [5]. Leur rupture est à l’origine d’un emphysème interstitiel cheminant le long des septas, rejoignant le médiastin par le hile et ou le ligament triangulaire puis les espaces sous cutanées cervicaux, péricardiques ou rétropéritoneaux. Les facteurs déclenchants sont les manœuvres de Valsalva, la toux, le travail chez la parturiente, les efforts de vomissements,une crise d’asthme, un exercice physique ,l’inhalation de cocaïne, une chimiothérapie (bléomycine), une acidocétose diabétique [1, 6, 7]. Dans Notre observation on note l’exacerbation de la crise d’asthme et l’effort de toux qui ont été incriminés dans la survenue du pneumomédiastin chez notre patient. La douleur est le principal symptôme. Elle est souvent en coup de poignard, augmentant avec la respiration et irradiant vers le cou. Elle peut être accompagnée de dyspnée, d’une modification de la voix secondaire à une irritation pharyngée accompagnée de toux et de dysphagie. A l’examen clinique on peut retrouver une crépitation neigeuse [8], témoignant d’un emphysème sous cutané ainsi que le signe de Hamman [9] qui définit par la présence de crépitements synchrones aux bruits du cœur à l’auscultation cardiaque. Bien que rare les complications de cette pathologie sont le pneumothorax et le pneumomédiastin sous tension. Le pneumothorax peut être lui-même compliqué d’une mise sous tension et d’une localisation bilatérale. Le pneumomédiastin sous tension diminuera en premier le retour veineux, avec possibilité de désamorçage de la pompe cardiaque et d’insuffisance cardiaque (tamponnade gazeuse). Le pneumomédiastin spontané non compliqué, est le plus souvent bénin. Ie traitement associe le repos, l’oxygénothérapie et les antalgiques .une surveillance clinique, ainsi qu’un monitoring cardio-respiratoire, sont souvent nécessaires quelques jours avant un contrôle de sortie par radiographie ou scanner thoracique. Le pneumomédiastin sous tension, doit quant à lui, être drainé. Le drainage se fera soit par insertion d’un cathéter au niveau du creux sussternal [10], soit par une petite médiastinostomie.

## Conclusion

Le pneumomédiastin spontané est une entité très rare et bénigne. Il doit être considéré dans le diagnostic différentiel d'une douleur thoracique brutale chez l'adolescent ou le jeune adulte asthmatique, légitimant la réalisation de clichés thoraciques au moindre doute clinique.
